# Lysine-Leucine-Rich Frog Skin Antimicrobial Peptides Inhibit Breast Cancer Metastasis by Reprogramming Tumor-Associated Macrophage Polarization

**DOI:** 10.3390/ijms26178627

**Published:** 2025-09-04

**Authors:** Zhenyan Li, Xuan Zhou, Weibing Dong, Ang Li

**Affiliations:** 1 School of Life Science, Liaoning Normal University, Dalian 116081, China; 2 School of Life Science, Hangzhou Institute for Advanced Study, University of Chinese Academy of Sciences, Hangzhou 310024, China; 3 School of Basic Medical Sciences, Dalian University of Technology, Dalian 116024, China

**Keywords:** tumor-associated macrophage, polarization, antimicrobial peptide, breast cancer, metastasis

## Abstract

Tumor-associated macrophages (TAMs) are one of the most important components of the tumor microenvironment and play a critical role in promoting tumor invasion and metastasis. These cells have become a new therapeutic target for inhibiting tumor progression. Lysine/leucine-rich antimicrobial peptides have well-documented anticancer activity in vitro, but their immune regulatory activity in human macrophages is not clear. The present study investigated the regulatory effects of lysine/leucine-rich peptides on the polarization of M2-like macrophages and the metastasis of breast cancer cells mediated by M2-like TAMs in the tumor microenvironment (TME). Our results revealed remarkable inhibition of the polarization of M2-like macrophages following treatment with lysine/leucine-rich antimicrobial peptides, which was accompanied by a significant reduction in the expression of the M2-like macrophage-specific factors interleukin-10 (IL-10) and transforming growth factor-β (TGF-β1) and the M2 macrophage-specific marker CD206. The lysine/leucine-rich antimicrobial peptides downregulated the expression of PPARγ and Krüppel-like factor 4 (KLF4) and the phosphorylation of STAT6 in the STAT6 signaling pathway, which resulted in a decrease in IL-10 and TGF-β1. Moreover, we found that lysine/leucine-rich antimicrobial peptide-treated macrophages reduced the migration of cancer cells by inhibiting the phosphorylation of the mTOR, smad2 and ERK proteins during tumor metastasis. These findings highlight the potential of lysine/leucine-rich antimicrobial peptides as therapeutic agents that target M2-like macrophages to inhibit cancer cell metastasis.

## 1. Introduction

Breast cancer is one of the most progressive malignant tumors affecting women worldwide and remains a main contributor to cancer-related deaths [1,2,3]. Despite recent advances in breast cancer treatment, such as chemotherapy, surgery, and radiation therapy, metastasis is difficult to treat in the clinic and seriously affects the prognosis of patients. Compared to other types of cancer, breast cancer is characterized by a large population of macrophages, which constitute approximately 50% of the tumor mass [4]. These macrophages, called TAMs, exist in a complex TME together with fibroblasts, dendritic cells, and lymphocytes and participate in the regulation of immunosuppression to promote resistance to immunotherapy [5,6]. TAMs exert significant immunosuppressive effects on tumor cell growth, metastasis, and progression in response to therapy and are currently receiving increasing attention in the clinical treatment of tumors [7,8].

TAMs are generally classified into two subpopulations, the M1-like phenotype and the M2-like phenotype. M1-like TAMs exhibit antitumor activity via the release of pro-inflammatory factors, including tumor necrosis factor-α (TNF-α), IL-1β, and IL-6, and immune activation. Conversely, M2-like TAMs play crucial roles in promoting tumor metastasis, invasion and drug resistance and generate immunosuppressive factors, including TGF-β and IL-10 [9,10]. In breast cancer, TAMs are generally characterized as M2 macrophages that are recruited and manipulated by cytokines, chemokines, and growth factors [11,12]. The cytokine IL-4 promotes M2-type macrophage polarization via the activation of the STAT6 and KLF4 signaling pathways [13,14]. IL-4 binds to the IL-4Rα receptor, which results in activation of the kinase JAK1/JAK3 and the phosphorylation of STAT6. Phosphorylated STAT6 enters the nucleus, which induces the activation of KLF4 and PPARγ, and the expression and release of M2-specific factors, such as IL-10, Arg-1 and TGF-β. The M2-specific marker CD206 is expressed on the cell surface [15,16]. The released extracellular TGF-β and EGF act on tumor cells to activate EMT, which increases cell invasion and metastasis and promotes tumor development and metastasis. TGF-β binds to type I and type II receptors on the cell membrane to form a ligand-receptor complex, which binds and phosphorylates Smad2/3 of the Smad protein family. This complex forms a complex with Smad4 in the cytoplasm and translocates to the nucleus, where it binds to other transcription factors to regulate the expression of specific genes that induce EMT [17,18]. EGF produced by M2-type TAMs binds to EGFR on the cell membrane and activates the downstream MEK/ERK and PI3K/AKT signaling pathways to recruit phosphorylated mTOR, thereby regulating the expression of specific genes and promoting EMT [19]. Some studies showed that the transcription factors STAT6 and KLF4 promoted IL-4-induced M2 polarization in vivo and in vitro. Therefore, there is a growing emphasis on the development of adjuvant anticancer therapies by repolarizing TAMs from the M2 phenotype to the pro-inflammatory M1-like phenotype. Recent studies have demonstrated that targeting the M2 polarization of TAMs within the TME is a promising potential therapeutic approach.

Antimicrobial peptides (AMPs) are typically short amino acid chains with a positive net charge that exhibit broad-spectrum antimicrobial activities, killing gram-negative and Gram-positive bacteria, viruses, fungi and parasites [20,21,22,23]. AMPs rapidly kill invading pathogens via initial interactions with the negatively charged outer and/or inner membranes of bacteria, but their amphipathic character causes membrane permeabilization and disruption, which induces leakage of cytoplasmic components and the death of microorganisms [24,25]. Notably, AMPs play important roles in host defense against pathogen infections and form valuable molecules in the innate immune system [26,27]. The peptides used in this study are part of a series of lysine/leucine-rich AMPs originally designed and synthesized based on the sequence of temporin-1CEb, a natural peptide isolated from the skin secretions of *Rana chensinensis* [28]. The design principles for such amphipathic, cationic α-helical peptides are supported by foundational work on model peptide systems, notably the studies by Lear and DeGrado in the 1980s, which established the relationship between primary sequence, secondary structure, and membrane activity for leucine-lysine-rich peptides [29]. Furthermore, the significant body of research on the designed lysine/leucine-rich peptide C18G, which exhibits potent antimicrobial and immunomodulatory activities [30], provides a strong precedent for the functional potential of peptides with this architecture. Among all of the peptides, L-K6, L-K6V1 and L-K6V2 have the same net positive charge (+7), but their hydrophobicity values (H) decrease from 11.6 to 10.1. L-K5 has the same amino acid sequence as L-K6 but lacks a Lys residue at the COOH terminus, and it has a greater H (12.3) than L-K6. These lysine/leucine-rich peptides adopt an α-helix in 50% trifluoroethanol/water and 30 mM SDS solutions and exhibit high antimicrobial activity against gram-positive and gram-negative bacteria and cytotoxicity against various cancer cells, with MCF-7 breast cancer cells being the most sensitive [31,32,33]. The amino acid sequences and key physicochemical characteristics of these peptides are detailed in Table 1. However, the immune regulatory activities of these AMPs and their analogs in human macrophages are not fully understood. The present study investigated the effects and possible mechanism of action of lysine/leucine-rich AMPs on the formation of TAMs in the TME and the metastasis of breast cancer cells induced by TAMs.

## 2. Results

### 2.1. The Lysine/Leucine-Rich Peptide L-K5 Exerts Antitumorigenic and Metastatic Effects on Breast Cancer Cells In Vivo

**Figure 1:**
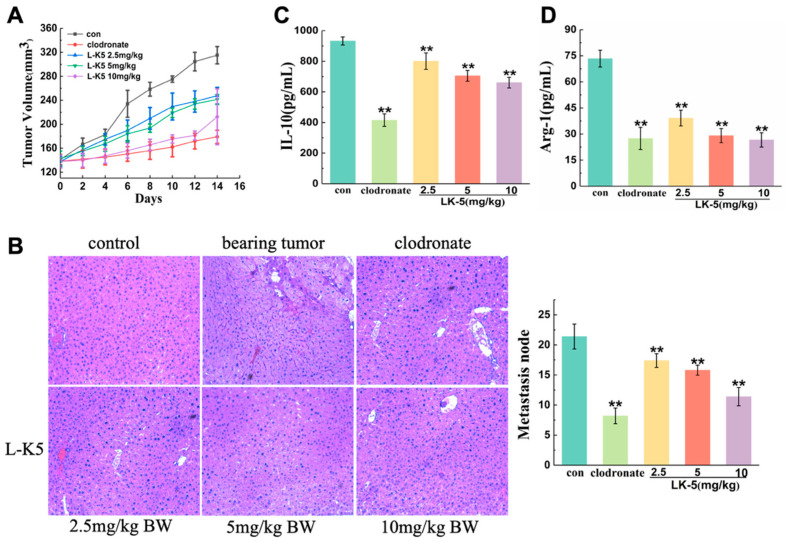
The lysine/leucine-rich peptide L-K5 has an inhibitory effect on mouse breast tumors. (**A**) Tumor volume of the mice. (**B**) Liver metastases by H&E staining; images were acquired at 100× magnification (10× objective lens) by Zeiss Axio Observer 7 microscope. (**C**,**D**) IL-10 and Arg-1 levels in mouse serum. The values presented are expressed as the means ± standard deviations (SDs) and are based on data obtained from three biologically independent samples. Compared to control: ** *p* < 0.01.

To investigate the anticancer effect of the lysine/leucine-rich peptide L-K5 in vivo, a subcutaneous breast tumor mouse model was established via the subcutaneous injection of MCF-7 cells. After 15 days of L-K5 local injection, the mice were sacrificed, the tumors were carefully excised, and the tumor volume was calculated. As shown in Figure 1A**,** the tumor volume was significantly lower in the L-K5 treatment group than the control group over time (*p* < 0.05). The tumor volume decreased 21.44%, 23.44% and 32.6% after treatment with L-K5 at doses of 2.5, 5 and 10 mg/kg, respectively. The positive control clodronate, a drug for the treatment of tumor metastases, decreased the tumor volume by 43.18%. To investigate the effect of L-K5 on liver metastasis, liver sections from breast tumor-bearing mice were stained with HE. The results revealed that the number of liver metastases was significantly lower in the L-K5 treatment group compared to the control group (*p* < 0.05, Figure 1B). Representative H&E-stained liver sections are shown in Figure 1B, where the dark purple metastatic foci are clearly visible in the control group but significantly reduced in number and size after L-K5 treatment. This histological observation quantitatively corresponds to the decreased count of metastatic points, confirming the inhibitory effect of L-K5 on breast cancer metastasis to the liver. The average number of metastatic points on the liver surface was 21 in the control group and 8 in the clodronate group, with an inhibition rate of 61.69%. The average numbers of metastatic points were 17, 16 and 11 in the L-K5 groups at 2.5, 5 and 10 mg/kg, respectively. The inhibition rates were 18.69%, 26.17% and 46.73%, respectively.

To determine whether the antitumor effect of L-K5 was associated with the regulation of macrophage polarization, we analyzed the release of IL-10, which is the specific cytokine of M2 macrophages, and Arg-1, which serves as a hallmark marker for M2 macrophages in mouse serum. As shown in Figure 1C,D, L-K5 significantly reduced the release of IL-10 and Arg-1 in a dose-dependent manner. Compared to the control, L-K5 at doses of 2.5, 5 and 10 mg/kg decreased the release of IL-10 by 14.14%, 24.42% and 29.2%, respectively, and decreased Arg-1 by 46.62%, 60.36% and 63.7%, respectively. Taken together, these results suggest that the lysine/leucine-rich peptide L-K5 exerted antitumor effects and suppressed breast cancer metastasis in vivo.

### 2.2. Lysine/Leucine-Rich Peptides Inhibit the Polarization of Human M2 Macrophages

**Figure 2:**
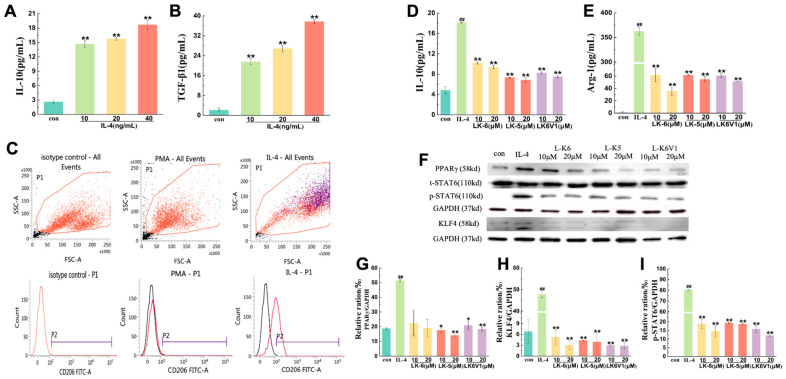
Regulatory effects of lysine/leucine-rich peptides on IL-4-induced M2-like macrophage polarization and the expression of signaling pathway proteins. (**A**,**B**) IL-4 induces the expression of IL-10 and Arg-1 in PMA-differentiated U937 macrophages. (**C**) IL-4 induces the expression of the M2-like macrophage marker CD206, as shown by flow cytometry. (**D**,**E**) Effects of lysine/leucine-rich peptides on the release of IL-10 and Arg-1 in IL-4-induced M2-like macrophages. (**F**–**I**) Western blot analysis revealed the effects of lysine/leucine-rich peptides on the expression of the signaling proteins PPAR-γ and KLF4 and the level of phosphorylated STAT6. The values presented are expressed as the means ± standard deviations (SDs) and are based on data obtained from three biologically independent samples. Compared to thee control: * *p* < 0.05, ** *p* < 0.01, ^##^ *p* < 0.0001.

Macrophages play important roles in the immune response and tissue homeostasis by demonstrating remarkable plasticity in response to microenvironmental cues [34]. In this study, we used PMA-differentiated human U937 monocytes (M0 macrophages) stimulated with IL-4 to generate a model of macrophage polarization. After U937 monocytes were incubated in the presence of PMA and became adherent and rounded, 10, 20 and 40 ng/mL IL-4 were used to induce macrophage differentiation into the M2 phenotype. As shown in Figure 2A,B, IL-4 increased the levels of IL-10 and TGF-β1 in a concentration-dependent manner. Compared to control, the release of IL-10 was 5.6, 6 and 7.1 times greater, and the release of TGF-β1 was 11.4, 13.5 and 18.7 times greater, respectively. CD206 is an important surface marker of M2-type macrophages. IL-4 (10 ng/mL) increased the expression of the recognized macrophage marker CD206 (M2 phenotype) by 43.8% (Figure 2C), which suggested that IL-4 successfully induced M2 macrophage polarization.

The cytotoxicity of three lysine/leucine-rich antimicrobial peptides to PMA-differentiated U937 macrophages was first detected using the MTT method. The results revealed that the cell viability was greater than 80% when the concentrations of L-K6, L-K5 and L-K6V1 were 10 and 20 μM (Figure S1A). Therefore, L-K6, L-K5 and L-K6V1 (10 and 20 μM) were used for subsequent in vitro studies.

To investigate the effects of the three lysine/leucine-rich antimicrobial peptides on IL-4-induced M2 polarization in macrophages, PMA-differentiated U937 cells were induced in the absence or presence of L-K6, L-K5 and L-K6V1 at concentrations of 10 μM and 20 μM for 24 h. The levels of IL-10 and Arg-1 in the cell supernatant were detected. As shown in Figure 2D,E, IL-4 significantly induced the release of the M2-type specific factors IL-10 and Arg-1 (*p* < 0.01). Compared to the control, the release of IL-10 and Arg-1 increased 3- and 417-fold, respectively. However, different concentrations of the three lysine/leucine-rich peptides significantly decreased the release of IL-10 and Arg-1 (*p* < 0.01). Compared to the IL-4 groups, 10 μM and 20 μM L-K5 reduced the IL-10 content by 59.6% and 62.3% and by 83.0% and 84.9% for Arg-1, respectively. L-K6 at concentrations of 10 and 20 μM reduced the IL-10 content by 43.9% and 48.3%, respectively, and by 82.9% and 90.2%, respectively, for Arg-1. L-K6V1 at concentrations of 10 and 20 μM reduced the IL-10 content by 54.5% and 58.7%, respectively, and by 83.2% and 85.8%, respectively, for Arg-1.

### 2.3. Lysine/Leucine-Rich Peptides Downregulated the STAT6-Related Signaling Pathway in Human Macrophages

Peroxisome proliferator-activated receptor γ (PPAR-γ) and signal transducer and activator of transcription 6 (STAT6), along with Krüppel-like factor 4 (KLF4), induce each other and promote M2 polarization in IL-4-induced macrophages [15,16]. To understand the effects of lysine/leucine-rich peptides on M2 polarization in PMA-differentiated U937 macrophages, the expression levels of STAT6 signaling pathway proteins were detected via Western blotting. The results revealed that IL-4 increased PPAR-γ expression by 2.5 times the control group, but the three lysine/leucine-rich peptides at concentrations of 10 and 20 μM significantly downregulated the expression of PPAR-γ (*p* < 0.05) (Figure 2F,G). L-K5 exhibited the most significant inhibitory effect, with inhibition rates of 63.3% and 70.6% at peptide concentrations of 10 and 20 μM, respectively. L-K6V1 (10 and 20 μM) downregulated the expression of PPAR-γ by 56.1% and 61.8%, respectively, and by 53.4% and 60.2%, respectively, in response to L-K6. As shown in Figure 2F,H, IL-4 increased KLF4 expression by 6.7-fold compared to the control. However, KLF4 expression was downregulated by approximately 80% in IL4-induced U937 macrophages treated with 10 and 20 μM of L-K6, L-K5 and L-K6V1. The expression level of phosphorylated STAT6 (*p*STAT6) was detected using Western blotting. We found that *p*STAT6 was not expressed in the control group, but *p*STAT6 was significantly increased after the addition of IL-4, which indicated that M2 macrophages had formed at this time (Figure 2F,I). The three lysine/leucine-rich peptides at concentrations of 10 and 20 μM significantly downregulated the expression of *p*STAT6 (*p* < 0.05). L-K5 at peptide concentrations of 10 and 20 μM reduced the phosphorylation of STAT6 by approximately 76%, and 10 and 20 μM L-K6 and L-K6V1 downregulated the expression of *p*STAT6 by 80–85%. Taken together, these three lysine/leucine-rich peptides downregulated the protein expression of the STAT6 signaling pathway to inhibit the development of IL-4-induced M2-type macrophage polarization.

### 2.4. Lysine/Leucine-Rich Peptides Suppress the Migration of MCF7 Cells by Regulating Macrophage M2 Polarization

**Figure 3:**
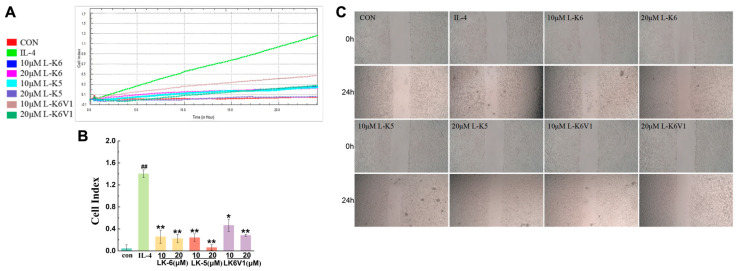
Lysine/leucine-rich peptides suppress the migration of MCF-7 breast cancer cells. (**A**) Dynamic detection of cell migration using RTCA. (**B**) Histogram of cell migration. (**C**) Scratch assay of MCF-7 cells treated with IL-4-induced M2-like macrophages in the absence or presence of 10 and 20 μM L-K6, L-K5 and L-K6V1 for 24 h. The values are presented as the means ± standard deviations (SDs) and are based on data obtained from three biologically independent samples. Compared to IL-4 group: * *p* < 0.05, ** *p* < 0.01, ^##^ *p* < 0.0001.

The above results revealed that lysine/leucine-rich peptides suppressed M2-type macrophage polarization. The effects of lysine/leucine-rich peptides on the migration of human breast cancer MCF-7 cells mediated by M2-type macrophages were investigated in a coculture system. First, the cytotoxic effects of the peptides on MCF-7 cells were assessed. The results revealed that the viability of MCF-7 cells was greater than 80% after treatment with 10 and 20 μM of L-K6, L-K5 and L-K6V1, which indicated the low cytotoxicity of the three peptides to MCF-7 cells (Figure S1B). Second, real-time cell analysis (RTCA) was used to evaluate the effects of the peptides on the migration of MCF-7 cells. PMA-differentiated U937 macrophages were treated with IL-4 in the absence or presence of 10 and 20 μM of L-K6, L-K5 and L-K6V1 for 24 h to induce the polarization of M2 macrophages. The various collected conditioned media were subsequently incubated with the MCF-7 cells in the RTCA pore plate, and the number of migrating cells was monitored in real time. As shown in Figure 3A,B, IL-4-induced macrophages promoted the migration of MCF-7 cells. However, the lysine/leucine-rich peptide-treated macrophages significantly reduced the migration of MCF-7 cells compared to the IL-4 group. The polarized M2 macrophages treated with 10 and 20 μM L-K5 reduced MCF-7 cell migration by 80.8% and 95.1%, respectively. Treatment with 10 and 20 μM L-K6 reduced migration by 79.5% and 82.1%, respectively, and treatment with 10 and 20 μM L-K6V1 reduced migration by 62.9% and 77.4%, respectively. Similarly, treatment with the three peptides markedly inhibited the migration of MCF-7 cells in the scratch assay (Figure 3C). These results suggested that the lysine/leucine-rich peptides suppressed macrophage-mediated tumor cell migration.

**Figure 4:**
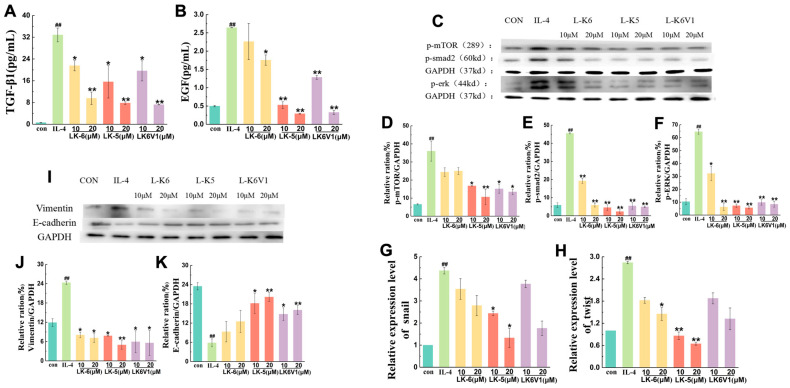
Lysine/leucine-rich peptides suppress the migration of breast cancer MCF-7 cells by downregulating the expression of signaling proteins. (**A**,**B**) Cytokines TGF-β and EGF are secreted by tumor cells. (**C**–**F**) Western blot analysis revealed the effects of lysine/leucine-rich peptides on the phosphorylation levels of the signaling proteins mTOR, Smad and ERK. (**G**,**H**) RT-qPCR was used to measure the mRNA levels of the *snail* and *twist* genes. (**I**–**K**) Western blot analysis revealed the effects of lysine/leucine-rich peptides on the phosphorylated levels of signal proteins vimentin and E-cadherin. The values presented are expressed as the means ± standard deviations (SDs) and are based on data obtained from three biologically independent samples. Compared to IL-4 group: * *p* < 0.05, ** *p* < 0.01, ^##^ *p* < 0.0001.

The cytokines TGF-β and EGF secreted by tumor cells are involved in the metastasis of tumor cells via the regulation of the Smad and MEK/ERK signaling pathways. To explore the mechanism by which lysine/leucine-rich peptides affect tumor cell migration by regulating M2 macrophage polarization, the levels of TGF-β and EGF were first detected in M2 macrophages treated with L-K6, L-K5 and L-K6V1. As shown in Figure 4A, the TGF-β1 level was more than 30 times greater in MCF-7 cells treated with IL-4-induced M2 macrophages compared to the control cells. However, the three peptides significantly downregulated the TGF-β1 level in a concentration-dependent manner (*p* < 0.05). L-K6, L-K5 and L-K6V1 at a concentration of 20 μM reduced the TGF-β1 levels by 70.9%, 76.3% and 78%, respectively. The EGF level increased 5-fold in the M2 macrophages induced by IL-4 compared to the control, and L-K6, L-K5 and L-K6V1 at a concentration of 20 μM reduced the EGF level by 30.3%, 88% and 87.7%, respectively (Figure 4B).

The protein levels of phosphorylated mTOR, Smad (a key component of the Smad signaling pathway), and ERK (a central kinase in the ERK signaling pathway) were detected using Western blotting. The results revealed a 3.9-fold increase in the level of phosphorylated mTOR (*p*mTOR) protein in MCF-7 cells treated with IL-4-induced M2 macrophages compared to the control, but the three peptides significantly downregulated *p*mTOR expression in a concentration-dependent manner (*p* < 0.05). Among the three peptides, L-K5 exhibited the best inhibition, with decreases of 54.7% and 73% when the L-K5 concentration was 10 and 20 μM, respectively (Figure 4C,D). Compared to no peptide treatment, L-K6V1 treatment downregulated *p*mTOR expression by 61.5% and 65.8%, respectively, and 10 and 20 μM L-K6 treatment downregulated *p*mTOR expression by 36.1% and 36.5%, respectively (Figure 4D). The expression level of phosphorylated Smad (*p*Smad) was upregulated 8.2-fold in MCF-7 cells treated with IL-4-induced M2 macrophages compared to the control. The three peptides significantly downregulated *p*Smad expression in a concentration-dependent manner (*p* < 0.05) (Figure 4E). As shown in Figure 4F, the expression level of phosphorylated ERK (*p*ERK) protein was upregulated 2.5-fold in MCF-7 cells treated with IL-4-induced M2 macrophages compared to the control. Treatment with L-K5, L-K6 and L-K6V1 at a concentration of 20 μM downregulated *p*ERK expression by 91.2%, 58.4% and 56.2%, respectively.

Epithelial-mesenchymal transition (EMT) plays an important role in tumor cell migration. The transcription factors Snail and Twist regulate EMT [35,36]. The mRNA levels of *snail* and *twist* were detected using RT-qPCR to explore the effects of the lysine/leucine-rich peptides on breast cancer cell metastasis. As shown in Figure 4G, *snail* mRNA expression was increased 4.3-fold in MCF-7 cells cultured with IL-4-conditioned medium compared to the control group. Treatment with 20 μM L-K5 and L-K6V1 reduced *snail* mRNA expression by 69.1% and 58.9%, respectively. The effects of the three peptide treatments on the mRNA expression of the *twist* gene were similar. Compared to the IL-4 group, *twist* mRNA expression was significantly lower in the peptide treatment group (Figure 4H). L-K5 (20 μM) had the greatest inhibitory effect (74.4%).

The expression of vimentin and E-cadherin was detected using Western blotting to further examine the effect of M2 TAMs treated with lysine/leucine-rich peptides because increased vimentin protein expression and decreased E-cadherin protein expression are the main markers of EMT. Western blot analysis revealed a marked increase in the expression of vimentin in MCF-7 cells following treatment with a conditioned medium from IL-4-induced M2 macrophages (*p* < 0.05). The protein expression was downregulated by approximately 65% after treatment with the three peptides compared to the IL-4-treated group (Figure 4I,J). In contrast, the protein expression of E-cadherin significantly decreased in MCF-7 cells treated with the conditioned medium of IL-4-induced M2 macrophages, but treatment with the three peptides significantly upregulated the expression of E-cadherin (*p* < 0.05). L-K5 (10 μM and 20 μM) increased the protein expression of E-cadherin by 66.3% and 69.7%, respectively (Figure 4K). These results suggested that the lysine/leucine-rich peptides reduced EMT by regulating the expression of the marker proteins vimentin and E-cadherin.

## 3. Discussion

Our study confirms the potent in vivo antitumor and anti-metastatic activity of the lysine/leucine-rich antimicrobial peptide L-K5 in a murine breast cancer model. We demonstrate that this effect is mechanistically linked to a novel immunomodulatory function: the reprogramming of Tumor-Associated Macrophages (TAMs). This is evidenced by the dose-dependent reduction in the serum levels of the IL-10 (M2-specific cytokines) and Arg-1 (hallmark marker for M2 macrophages) following L-K5 treatment (Figure 1C,D), which correlated with inhibited tumor growth and reduced liver metastasis (Figure 1A,B).

Building on this in vivo finding, our in vitro investigations revealed that L-K5 and its analogs (L-K6, L-K6V1) directly inhibit the IL-4-induced M2 polarization of human macrophages. This effect was not merely phenotypic but was achieved through the suppression of the core IL-4R/STAT6 signaling axis, as demonstrated by the significant decrease in STAT6 phosphorylation and the downregulation of its downstream transcription factors, PPAR-γ and KLF4 (Figure 2F–I). The subsequent attenuation in the production of key M2 cytokines (IL-10, Arg-1; Figure 2D,E) provides a direct mechanistic rationale for the observed in vivo effects. In contrast to the primarily direct cytotoxic effects against cancer cells reported in previous studies, our work provides a more nuanced understanding of L-K5’s dual role by highlighting its capacity to modulate the tumor immune microenvironment through TAM reprogramming. As canonical cationic amphipathic peptides, L-K5 and its analogs possess inherent membrane-active potential. However, the observed low cytotoxicity at effective concentrations (10–20 μM) suggests that the profound inhibition of M2 polarization is not a consequence of nonspecific membrane lysis but rather of specific immunomodulatory reprogramming. Our data indicate that this occurs primarily through the suppression of the IL-4R/STAT6 signaling axis.

M2-like macrophages are one of two subpopulations of tumor-associated macrophages (TAMs). TAMs are profoundly important for tumor therapy. TAMs play an important role in various aspects of tumor progression and treatment response [5,37]. TAMs represent a complex network comprised of diverse cellular and noncellular components that interact dynamically with tumor cells. Typically, TAMs exhibit two distinct polarization states, the M1 type and the M2 type. M1 macrophages secrete pro-inflammatory cytokines, such as TNF-α, to inhibit tumor growth, whereas M2-like TAMs produce immunosuppressive factors, including transforming growth factor–β (TGF-β) and interleukin-10 (IL-10), and play crucial roles in promoting tumor metastasis, invasion and drug resistance [10,38]. IL-4 and other cytokines promote M2-type macrophage polarization. To investigate the effects of three lysine/leucine-rich AMPs, L-K6, L-K5 and L-K6V1, on IL-4-induced M2 polarization of macrophages, PMA-differentiated U937 cells were induced with IL-4 in the absence or presence of L-K6, L-K5 and L-K6V1 at concentrations of 10 μM and 20 μM for 24 h. The levels of IL-10 and Arg-1 in the cell supernatant were detected. Our results revealed that the three lysine/leucine-rich peptides significantly decreased the release of IL-10 and Arg-1 (*p* < 0.01), which indicated the suppression of M2-like TAM polarization.

IL-4 promotes M2-type macrophage polarization via activation of the STAT6 and KLF4 signaling pathways. The lysine/leucine-rich AMPs L-K6, L-K5 and L-K6V1 downregulated the expression of the PPAR-γ and KLF4 proteins and decreased the expression level of phosphorylated STAT6, which indicated that the three lysine/leucine-rich peptides downregulated the protein expression of the STAT6 signaling pathway to inhibit the formation of IL-4-induced M2-type macrophage polarization. This suppression of upstream STAT6 phosphorylation and the subsequent downregulation of the key transcription factors PPAR-γ and KLF4 provides the direct mechanistic rationale for the observed decrease in the expression of characteristic M2 markers, including IL-10 and Arg-1. These results revealed that lysine/leucine-rich peptides suppressed M2-type macrophage polarization. Here, the effects of lysine/leucine-rich peptides on the migration of human breast cancer MCF-7 cells mediated by M2-type macrophages were investigated in a coculture system. IL-4-induced macrophages promoted the migration of MCF-7 cells. However, lysine/leucine-rich peptide-treated macrophages significantly reduced the migration of MCF-7 cells. The cytokines TGF-β and EGF secreted by TAMs are involved in the metastasis of tumor cells via regulation of the Smad and MEK/ERK signaling pathways [39,40]. In the Smad pathway, TGF-β binds to type I and type II receptors on the cell membrane to form a ligand-receptor complex, which binds and phosphorylates Smad2/3 of the Smad family of proteins. This complex forms a complex with Smad4 in the cytoplasm and translocates to the nucleus, where it binds to other transcription factors to regulate the expression of specific genes. However, Smad-independent pathways are also involved in the TGF-β-induced EMT process, such as the P13K/AKT, MEK/ERK, JNK, and P38 pathways. Our results revealed that the three lysine/leucine-rich peptides significantly downregulated the TGF-β1 and EGF levels in a concentration-dependent manner (*p* < 0.05). Furthermore, the three lysine/leucine-rich peptides significantly downregulated the expression of phosphorylated ERK (a key kinase in the MAPK/ERK pathway), mTOR (a central regulator of the PI3K/AKT/mTOR pathway), and Smad (a primary component of the TGF-β/Smad pathway) in MCF-7 cells treated with IL-4-induced M2 macrophages, which suggests that lysine/leucine-rich AMPs suppress the phosphorylation of signal proteins in the Smad and ERK signaling pathways in MCF-7 cells to decrease the release of TGF-β and EGF cytokines.

In addition to various aspects of tumor progression and the treatment response of TAMs, EMT plays an important role in tumor cell migration. The transcription factors snail and twist regulate EMT [19,41,42]. Here, after treatment with lysine/leucine-rich AMPs, the mRNA levels of the *snail* and *twist* genes were significantly reduced in MCF-7 cells cultured with IL-4-induced conditioned medium. The results indicated that the expression of vimentin was downregulated, but the expression of E-cadherin was upregulated, which suggested that the lysine/leucine-rich peptides reduced EMT by regulating the expression of the marker proteins vimentin and E-cadherin. Several studies indicated that lysine/leucine-rich peptides modulated multiple intracellular cytokines within tumor cells.

Based on these results, we hypothesized that lysine/leucine-rich peptides suppress the growth of breast tumors and reduce liver metastasis by inhibiting the polarization of M2-like TAMs.

## 4. Materials and Methods

### 4.1. Peptide Synthesis

Peptides were synthesized by GL Biochem Ltd. (Shanghai, China) using standard Fmoc solid-phase technology (purity > 95%). The relative mass of the peptide was determined using MALDI-TOF MS (Shimadzu, Kyoto, Japan). The amino acid sequences and physical characteristics of the peptides are shown in Table 1.

### 4.2. Cell Lines and Cell Culture

The human myeloid leukemia cell line U937 and the human breast cancer cell line MCF7 were purchased from the Cell Bank of the China Science Academy (Shanghai, China). U937 cells and MCF-7 cells were cultured in RPMI-1640 supplemented with 10% fetal calf serum in a humidified atmosphere of 5% CO_2_ at 37 °C.

### 4.3. Animal Model

Fifty female BALB/c nude mice (6 weeks old, 18 ± 2 g) were purchased from GemPharmatech Co., Ltd. (Changzhou, China). First, a tumor-bearing mouse model was established via the subcutaneous injection of MCF-7 cells (2 × 10^6^ cells/mouse) into the right side of the back of each mouse. The tumor size was measured daily using a digital caliper and calculated according to the following formula: tumor size (V) = length × width × width/2. A tumor size of 100–150 mm^3^ indicated a complete tumor-bearing mouse model. The tumor-bearing mice were then randomly divided into five groups: the control group, the positive control group (clodronate) and the AMP L-K5 groups, with 10 mice in each group. L-K5 was dissolved in 0.9% saline and administered intraperitoneally daily at doses of 2.5, 5 and 10 mg/kg body weight for 14 consecutive days. The control animals received equal volumes of normal saline. The body weight, tumor size, activity and mental status of the mice in each group were observed and recorded daily. On day 15, the animals were sacrificed after anesthesia, and plasma was collected from the mice in each group for the determination of the cytokines IL-10 and Arg-1. The tumors were removed and immediately weighed. Mouse livers were subjected to HE staining.

### 4.4. Hematoxylin and Eosin Staining

Mouse livers were fixed in 4% paraformaldehyde in phosphate-buffered saline and embedded in paraffin. Each sample was cut into 4-μm-thick sections. The sections were stained with hematoxylin and eosin (HE, Sigma-Aldrich, St. Louis, MO, USA). Finally, photographs were taken using a Zeiss Axio Observer 7 microscope (Zeiss, Oberkochen, Germany) with an Image-Pro Plus medical image analysis system.

### 4.5. Cytotoxicity Assay

Peptide cytotoxicity against U937 cells and MCF-7 cells was determined using 3-(4,5-dimethylthiazol-2-yl)-2,5-diphenyltetrazolium bromide (MTT) assays. In brief, cells (5 × 10^5^ cells/well) were plated in 96-well plates and cultured overnight at 37 °C with 5% CO_2_. Following this incubation, the cells were treated with the peptides at concentrations of 10 and 20 μM for 24 h. A volume of 10 μL of 5 mg/mL MTT solution was added to each well, and the cells were incubated for an additional 4 h at 37 °C. Afterward, 150 μL of DMSO was added to dissolve the purple-blue MTT formazan precipitant. The absorbance was detected at 490 nm using a Multiskan FC microplate reader (Varioskan Flash Microplate Reader, Thermo Fisher Scientific Co., Beijing, China). Cell viability was calculated as follows: (OD_treated group_ − OD_Blank_)/(OD_control group_ − OD_Blank_) × 100%. The experiments were performed in triplicate, and the results are expressed as a percentage of inhibition in viable cells.

### 4.6. M2 Macrophage Polarization

To obtain U937-derived macrophages, U937 cells (5 × 10^5^ cells/well) were plated in 96-well plates and incubated with 100 ng/mL phorbol 12-myristate 13-acetate (PMA) at 37 °C for 48 h to permit adherence. After washing with fresh medium three times, the adherent U937-derived macrophages were cultured with 10 ng/mL interleukin-4 (IL-4) for an additional 24 h to allow differentiation into M2-polarized macrophages.

### 4.7. Cytokine Detection

U937-derived macrophages were induced by IL-4 into M2-polarized macrophages. After washing three times with phosphate-buffered saline (PBS), the supernatants were collected via centrifugation at 1000 rpm, and the production of TGF-β1, IL-10 and Arg-1 was quantified using a QuantiCyto human enzyme-linked immunosorbent assay (ELISA) kit (Neobioscience, Shanghai, China) according to the manufacturer’s instructions. Cells treated with medium alone, or IL-4 alone were used as negative controls for basal cytokine levels and positive controls, respectively. The data were analyzed based on three independent experiments in each group.

In a separate experiment designed to investigate potential intracellular effects, U937-derived macrophages were first treated with L-K6 and its analogs for 1 h, washed three times with phosphate-buffered saline (PBS) to remove extracellular peptides, and then incubated with IL-4 for 24 h to allow differentiation into M2-polarized macrophages. The levels of secreted TGF-β1, IL-10 and Arg-1 in the supernatants were measured using an ELISA kit. Data from this specific pre-treatment protocol are not presented in the figures but were consistent with the inhibitory effects observed in the co-treatment protocol.

### 4.8. Flow Cytometry

U937-derived macrophages were induced by IL-4 into M2-polarized macrophages. After washing three times with PBS, the cells were digested with 0.25% trypsin and collected via centrifugation at 500 rpm for 5 min. The cells were then stained with antibodies against CD206 for 45 min at 4 °C in the dark, washed twice and resuspended in PBS. The cells were sorted using a FACSCalibur cytometer (BD Biosciences, San Jose, CA, USA).

### 4.9. Conditioned Medium Preparation

U937-derived macrophages were incubated with IL-4 in the absence or presence of 10 or 20 μM antimicrobial peptides L-K6, L-K5 or L-K6V1 for 24 h. The polarized M2 macrophages were incubated in serum-free medium for 24 h. After centrifugation at 1000 rpm for 5 min, the supernatants were collected as conditioned media and stored at −80 °C.

### 4.10. Transwell Invasion Assay

Transwell invasion assays were performed in a 12-well cell culture chamber with Corning, Inc. Transwell chambers were used for xCELLigence real-time cellular analysis (ACEA Biosciences, San Diego, CA, USA). A total of 165 μL of serum-containing medium was added to the lower Transwell chamber, and a smooth meniscus with a convex surface was formed. A sensor plane of the lower Transwell chamber was placed relative to the upper chamber to avoid the pressure generated by the bubbles from interfering with the experimental results. Thirty microliters of serum-free medium were added to the upper chamber and incubated for 1 h in a CO_2_ incubator at 37 °C. MCF-7 cells (5 × 10^4^ cells/well) were treated with various collected conditioned media for 24 h. After baseline measurement, 100 µL of the cell suspension was added to the upper chamber and incubated at room temperature for 30 min. The system automatically scanned the RTCA pore plate in the RTCA station, and the detection was performed for 24 h once every 10 min.

### 4.11. Scratch Wound-Healing Migration Assay

MCF-7 cells (2 × 10^5^ cells/well) were seeded in a 12-well plate, and two lines were scraped from the cells using a sterile plastic pipette tip when the culture reached 80–90% confluence. After washing twice with warm serum-free medium to remove cellular debris, the cells were treated with various collected conditioned media for 24 h. Digital images were obtained using a Zeiss Observer 7 microscope (Zeiss, Germany), and cell migration was analyzed according to the percentage of the wound closure area using ImageJ software (ImageJ 1.8.0).

### 4.12. Real-Time Quantitative PCR (RT-qPCR)

Total RNA from cells was extracted using TRIzol reagent (Sigma, Shanghai, China), and cDNA was amplified via reverse transcription using a HiScript^®^ II Q RT SuperMix for qPCR (+gDNA wiper) kit (Vazyme, Nanjing, China) according to the manufacturer’s protocol. The primer sequences used for RT-qPCR are listed in Table 2. The relative RNA expression levels were quantified using RT-qPCR on an ABI-Prism 7500 Fast sequence detection system (Applied Biosystems, Foster City, CA, USA). PCR consisted of 40 cycles at 95 °C for 5 s, 60 °C for 30 s, and 70 °C for 30 s. The relative mRNA expression levels were analyzed using the 2^−∆∆CT^ method and LightCycler^®^ 96 software (Roche Diagnostics, Mannheim, Germany). GAPDH was used for normalization.

### 4.13. Western Blot Analysis

The cells were lysed in RIPA lysis buffer containing a mixture of PMSF and protease inhibitor. The cell lysates were collected via centrifugation at 12,000 rpm and 4 °C for 5 min then quickly frozen. The protein concentration was measured using a BCA protein assay kit (Beyotime, Shanghai, China). Proteins were separated on a 10% sodium dodecyl sulfate polyacrylamide gel (SDS-PAGE) and electroblotted onto a polyvinylidene fluoride (PVDF) membrane for 90 min. Five percent nonfat milk in TBST (20 mM Tris pH 7.5, 150 mM NaCl, and 0.1% Tween 20) was used to block the PVDF membranes for 2 h at room temperature. The membranes were incubated with primary antibodies against PPAR-γ, KLF4, E-cadherin, phosphorylated STAT6, phosphorylated ERK, phosphorylated mTOR, phosphorylated smad2 and vimentin overnight at 4 °C. After washing three times with TBST, the blots were incubated with a 1:1000 dilution of horseradish peroxidase-conjugated secondary antibodies (1:5000) at room temperature for 2 h, followed by an ECL chemiluminescence substrate (Thermo Scientific, Carlsbad, CA, USA) to develop the immunoreactivity. Proteins were detected using an Azure c500 imaging system (Azure Biosystems, Dublin, CA, USA) and quantified using ImageJ software (ImageJ 1.8.0). GAPDH was used as an internal control for normalization.

### 4.14. Statistical Analysis

The data are expressed as the means ± standard errors of the means (SEMs). The statistical significance of the differences was tested using Student’s *t*-tests. A *p*-value < 0.05 was considered statistically significant (* *p* < 0.05, ** *p* < 0.01).

## 5. Conclusions

In conclusion, this research presents groundbreaking evidence that three lysine/leucine-rich AMPs, L-K6, L-K5 and L-K6V1, efficiently promoted the polarization of M2 TAMs toward the M1 phenotype, which resulted in the suppression of tumor cell migration. These discoveries have substantial implications for the advancement of innovative therapeutic approaches and provide a fresh outlook for individuals suffering from cancer.

## Figures and Tables

**Table 1:** Physiochemistry characteristics of lysine/leucine-rich AMPs, the net charge and hydrophobicity were calculated using established methods [28].

Peptide	Amino Acid Sequence	MW (Da)	Net Charge	Hydrophobicity
L-K6	IKKILSKIKKLLK-NH_2_	1552	+7	11.6
L-K5	IKKIVSKIKKLL-NH_2_	1410	+6	12.3
L-K6V1	IKKIVSKIKKLLK-NH_2_	1538	+7	10.9

**Table 2:** Gene primer sequences.

Primer	Sequence	Gene ID
*gapdh* Forward	5′-GCCAAAAGGGTCATCATCTC-3′	NM_002046.3
*gapdh* Reverse	5′-GTAGAG GCAGGGATGATGTTC-3′	
*snail* Forward	5′-GGCTCCTTCGTCCTTCTCCTCTAC-3′	NM_005985
*snail* Reverse	5′-CCAGGCTGAGGTATTCCTTGTTGC-3′	
*twist* Forward	5′-CACCATCCTCACACCTCTGCATTC-3′	NM_000474
*twist* Reverse	5′-GGCTGATTGGCACGACCTCTTG-3′	

## Data Availability

The datasets presented in the study are included in the article/Supplementary Materials.

## Supplementary Materials

The following supporting information can be downloaded at: https://www.mdpi.com/article/10.3390/ijms26178627/s1.
