# Supplementary figures and images for: Lysine-Leucine-Rich Frog Skin Antimicrobial Peptides Inhibit Breast Cancer Metastasis by Reprogramming Tumor-Associated Macrophage Polarization
Source: Int J Mol Sci. 2025 Sep 4;26(17):8627. doi: 10.3390/ijms26178627 (PMC12429139; doi:10.3390/ijms26178627)

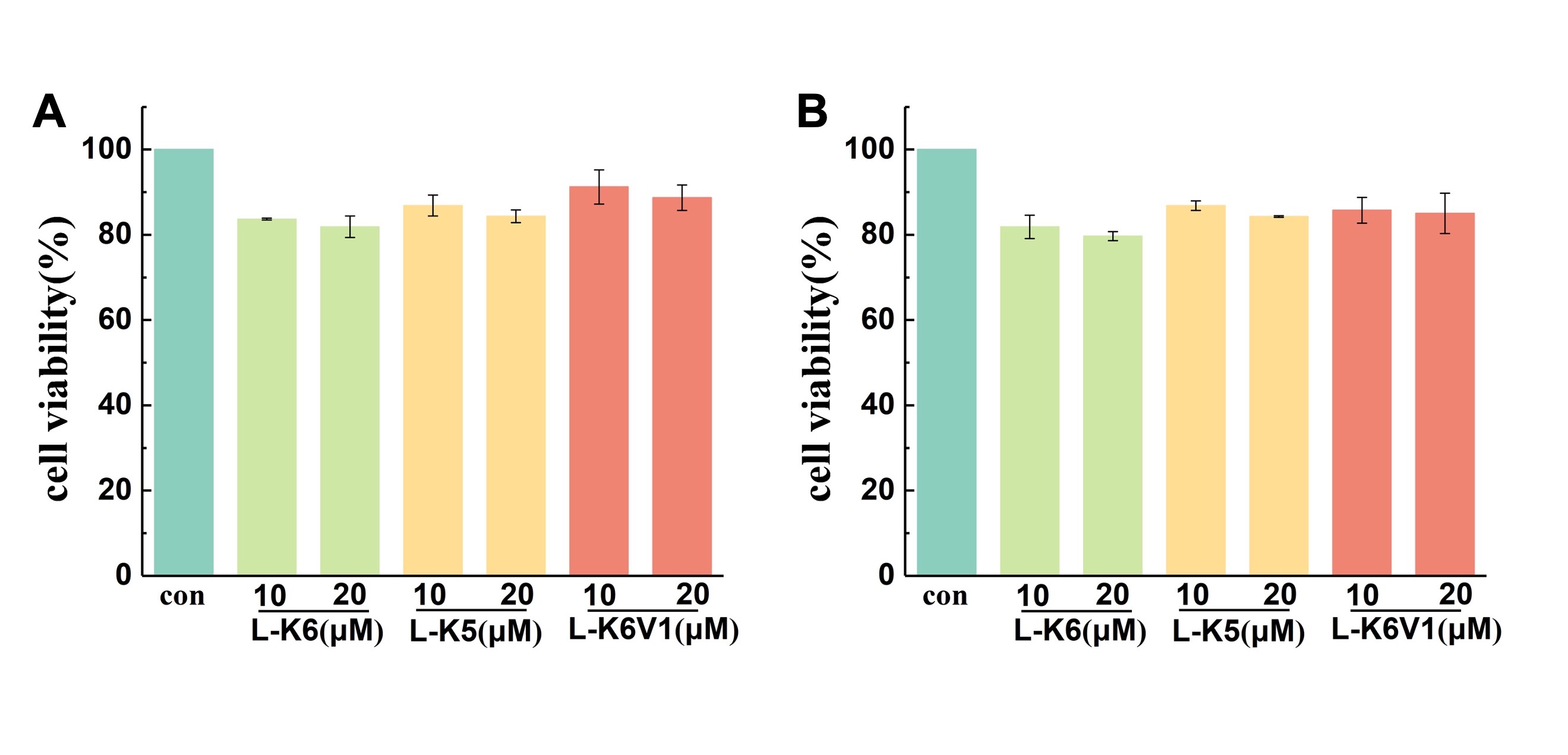

Supplement: Supplementary file 1 [file ijms-26-08627-s001.zip › ijms-3824900-supplementary/Figure S1.jpg]
